# Chromosome mapping of a Tc1-like transposon in species of the catfish *Ancistrus*

**DOI:** 10.3897/CompCytogen.v11i1.10519

**Published:** 2017-01-20

**Authors:** Keteryne Rodrigues da Silva, Sandra Mariotto, Liano Centofante, Patricia Pasquali Parise-Maltempi

**Affiliations:** 1 Laboratório de Citogenética Animal – Universidade Estadual Paulista “Júlio de Mesquita Filho” Campus de Rio Claro – Av 24A, 1515 Jardim Bela Vista- 13600-000- Rio Claro/SP, Brasil; 2 Instituto Federal de Ciências e Tecnologia do Mato Grosso, campus de Cuiabá – Bela Vista, MT, Brasil; 3 Instituto de Biociências, UFMT Universidade Federal de Mato Grosso, Cuiabá, MT, Brasil

**Keywords:** Repetitive DNA, enzyme digestion, chromosomal mapping, transposable elements, *in situ* fluorescence hybridization

## Abstract

The Tc1 mariner element is widely distributed among organisms and have been already described in different species of fish. The genus *Ancistrus* (Kner, 1854) has 68 nominal species and is part of an interesting taxonomic and cytogenetic group, as well as presenting a variation of chromosome number, ranging from 2n=34 to 54 chromosomes, and the existence of simple and multiple sex chromosome system and the occurrence of chromosomal polymorphisms involving chromosomes that carry the nucleolus organizer region. In this study, a repetitive element by restriction enzyme, from *Ancistrus* sp.1 “Flecha” was isolated, which showed similarity with a transposable element Tc1-mariner. Its chromosomal location is distributed in heterochromatic regions and along the chromosomal arms of all specimens covered in this study, confirming the pattern dispersed of this element found in other studies carried out with other species. Thus, this result reinforces the hypothesis that the sequence AnDraI is really a dispersed element isolated. As this isolated sequence showed the same pattern in all species which have different sex chromosomes systems, including in all sex chromosomes, we could know that it is not involved in sex chromosome differentiation.

## Introduction

The genome of eukaryotes consists mostly of large amounts of repetitive DNA, which has been associated with several functions in the genome, as can be seen in the review carried out by Shapiro (2010). These functions range from important roles in the structure of chromosomes, the telomere and centromere maintenance mechanism (Pardue and Debaryshe 2003, Wong and Choo 2004), involvement in DNA replication process (Li et al. 2002), of recombination (Biet et al. 1999) and gene expression (Liu et al. 2001, Peaston et al. 2004, Han and Boeke 2005, Volff 2006), in origin and evolution of sex and supernumerary chromosomes (Lyon 2000, Steinemann and Steinemann 2005, Parise-Maltempi et al. 2007), besides being used as important markers for cytogenetic studies of evolution, genome organization and identification of chromosomal rearrangements in several groups of organisms (Biémont and Vieira 2006, Martins 2007, Oliveira et al. 2013).

Basically, the repetitive sequences are represented by tandem repeats, as satellite DNA, minisatellite, and microsatellite repeats or dispersed along the DNA as retrotransposons and transposons (Charlesworth et al. 1994). The transposable elements (TEs) are classified according to the type of intermediate transposition, being of class I those that possess RNA intermediates and class II those whose intermediates are DNA molecules (Kidwell 2002). Transposons, belonging to Class II, representing most of the moderately repeated sequences of the eukaryotic genome, can be located in the region of constitutive heterochromatin and / or interspersed through the chromosomes and evolved through the ability to replicate making copies of themselves and moving to other regions of the genome (Capriglione et al. 2002).

When transposed, if the transposition occurs within promoter regions, introns or untranslated regions, it can affect the expression of this gene (Maksakova et al. 2006) and, although most of these mutations are harmful, the transposition of these elements have contributed to diversification of species due to generation of new alleles (Kapitonov and Jurka 2006). Its ability to spread in multiple copies may be regarded as a driving force for the evolution of the genome and, indeed seems to promote the variability of the genome, which may lead to a determination regulatory mutations and chromosomal rearrangements (Syvanen 1984, Charlesworth et al. 1994).

Based on the similarity between the sequences and phylogenetic analysis of the transposase, the transposable elements can be classified in ten families: Tc1/mariner, haT, P element, MuDR/Fokdback, Cacta, PiggyBac, Pif/Harbinger, Merlin, Transib and Banshee (Feschotte and Pritham 2007). Since the discovery of transposable elements in eukaryotes, elements such as Tc1 / mariner have been isolated from different fish species (Radice et al. 1994, Izsvák et al. 1995, Ivics et al. 1996, Capriglione et al. 2002, Krasnov et al. 2005, Pocwierz-Kotus et al. 2007, Liu et al. 2009).

This element, belonging to a superfamily of transposons, presents 1000 up to 2000 bp (Kidwell 2002), characterized by a simple structure with two inverted terminal repeats (TIRs) of approximately 28 bp. Also has an ORF (Opening Read Frame) encoding the transposase (Wallau et al. 2011) and is widely distributed among organisms, from protozoa to vertebrates. However, due to various events - mutations, deletions and insertions which become permanent component of the genome (Pocwierz et al. 2007), the majority is currently in an inactive form (Miskey et al. 2005).

The genus *Ancistrus* (Kner, 1854) is one of the most diverse of tribe Ancistrinae, popularly known as “cascudos”, and currently has 68 nominal species (Eschmeyer 2015). Its taxonomy is very confusing and a lot of species already have to be described. Based on chromosomal analysis, (Mariotto et al. 2011) suggested the existence of 13 cytotypes for the *Ancistrus* species found in the basis of rivers Paraguay, Araguaia-Tocantins and the Amazon in the Mato Grosso state (Brazil). They also suggested the existence of possible new species in this region, which show variation in chromosome number diploid ranging from 2n=34 to 54 chromosomes, presence of simple and multiple sex chromosome systems with both heterogametic sex and occurrence of chromosomal polymorphisms involving the chromosomes carrying the nucleolus organizing region for the group (Alves et al. 2003, Alves et al. 2006; Mariotto and Miyazawa 2006, Oliveira et al. 2007, Mariotto et al. 2009). Systems of ZZ/ZW and XX/XY sex chromosomes were found in populations of Ancistrus cf. dubius and *Ancistrus* sp 08 from the wetland of Mato Grosso state (Brazil) (Mariotto et al. 2004, Mariotto and Miyazawa 2006), the X0 system in *Ancistrus* n. sp.1 from “Rio Vermelho” located in Goiás state (Brazil) (Alves et al. 2006) and multiple systems of XX/XY1Y2 and Z1Z1Z2Z2/Z1Z2W1W2 for the species *Ancistrus* sp.1 “Balbina” and *Ancistrus* sp.2 “Barcelos” from Amazon state (Brazil) (De Oliveira et al. 2008).

Thus, taking into account the karyotype diversity of *Ancistrus*, including different sex chromosome systems, location of nucleolus organizer regions (NOR) and number of chromosomes, this study aimed to isolate repetitive sequences that could help in better understanding of the karyotype organization of the *Ancistrus* species.

## Material and methods

### Samples

The species of *Ancistrus* covered in this study were collected in the Flecha river, Creek Currupira, Pari and Sangradouro in the Paraguay river basin (Table 1). The collected material was taken to the Animal Genetics Laboratory at the Federal University of Mato Grosso, where 109 chromosome preparations were obtained.

**Table 1.** Collection site and number of species collected.

Specie	Collection site	Number of collected species
*Ancistrus* sp1 “Flecha”	15°58'7"S 57°19'7"W	18 F – 6 M
*Ancistrus* sp “Currupira”	15°7'59"S 56°49'47"W	19 F – 23 M
*Ancistrus* sp “Pari”	15°36'6"S 56°12'19"W	7 F – 12 M
*Ancistrus* sp “Sangradouro”	16°4'25"S 57°40'31.1"W	5 F – 4 M

### Preparation of mitotic chromosomes

The chromosome preparations were made from the kidney of specimens collected following the methodology described by Foresti et al. (1993). The material was stored in a freezer at -20 °C.

### Characterization of karyotypes

C-bands were detected according to Sumner (1972) to assemble the karyotypes.

### Obtaining repetitive sequences

The extraction of genomic DNA was performed from liver and fin of the specimens collected, basically following the protocol phenol / chloroform / isoamyl alcohol by Sambrook and Russel (2001). The extracted genomic DNA was digested with various restriction enzymes to isolation of repetitive sequences in a proportion of 30 μl DNA (100 ng) in 3 μl of enzyme. This solution was left at 37 °C (temperature according to the enzyme used) overnight and after 7 hours of digestion was further added 3 μl of enzyme. For precipitation and purification of the digested DNA 2 μl of 5M NaCl and 200 µL of ice cold 100% ethanol was added. This solution was stored for two hours at -80 °C and centrifuged after two hours. Digested DNA was eluted in 10 μl of distilled water and analyzed in agarose gel 1% stored in the freezer for later use. The bands of potential repetitive sequences were then purified according the QIAquick PCR Purification Kit protocol (Qiagen). To perform the cloning, competent bacteria were prepared in the laboratory according to chemical transformation with CaCl_2_ (Mandel and Higa 1970). The DNA fragments were inserted into plasmid vectors with pMOS Blue Kit (Amersham Biosciences) following the manufacturer’s specifications.

### DNA sequencing

The amplified and purified DNA by treatment with ExoSAP enzyme (USB) was sequenced by the method of Sanger et al. (1977) through outsourcing of services by MacroGen company (Korea). The editing of the sequences was performed on the program BioEdit sequence alignment editor v7.0.5.3 (Hall 1999) using the Clustal W tool for performing alignment of the sequences. For the characterization of the tools sequences were used: BLAST - Basic Local Alignment Search Tool at National Center for Biotechnology Information (NCBI) website (http://www.ncbi.nlm.nih.gov/blast); CENSOR; RepeatMasker, website (http://www.repeatmasker.org/cgi-bin/WEBRepeatMasker) and ORF Finder, the National Center for Biotechnology Information (NCBI) website (http://www.ncbi.nlm.nih.gov/gorf/gorf.html).

### DNA amplification via PCR

The recombinant clones were subjected to PCR (Polymerase Chain Reaction) for amplification using the universal primers M13 F - GTA AAA CGA CGG CCA G and M13 R - CAG GAA ACA GCT ATG AC under the following conditions: denaturation at 95 °C for 3 minutes, 34 cycles of denaturation at 95 °C for 30 seconds, annealing at 50 for 1 minute, 72 °C extension for 2 minutes and elongation at 72 °C for 5 minutes.

After sequencing, the divergent primers were designed KD7116F – TCA CAA CAC ACG TTT GTG GA and KD7116R – AGA GCA GGC TTT GAA TCG G manually, which was synthesized by SIGMA company. Subsequently, the amplification of the sequence with the primer KD7116-1 also in other possible different species from other populations was performed following conditions: denaturation at 94 for 5 minutes, 30 cycles of denaturation at 94 °C for 30 seconds, annealing at 58 °C for 1 minute 72 °C extension for 1 min and elongation at 72 °C for 7 minutes.

### Fluorescence *in situ* hybridization

*In situ* hybridization was performed following the protocol by Pinkel et al. (1986) with some modifications. The fluorescent probes were labeled with digoxigenin by nick translation. The slides were mounted with antifading solution containing DAPI and chromosomes observed using an Olympus BX51 microscope and digital camera model D. The images were captured using the DP Controller software.

## Results

### Karyotype analysis

**Figure 1.**
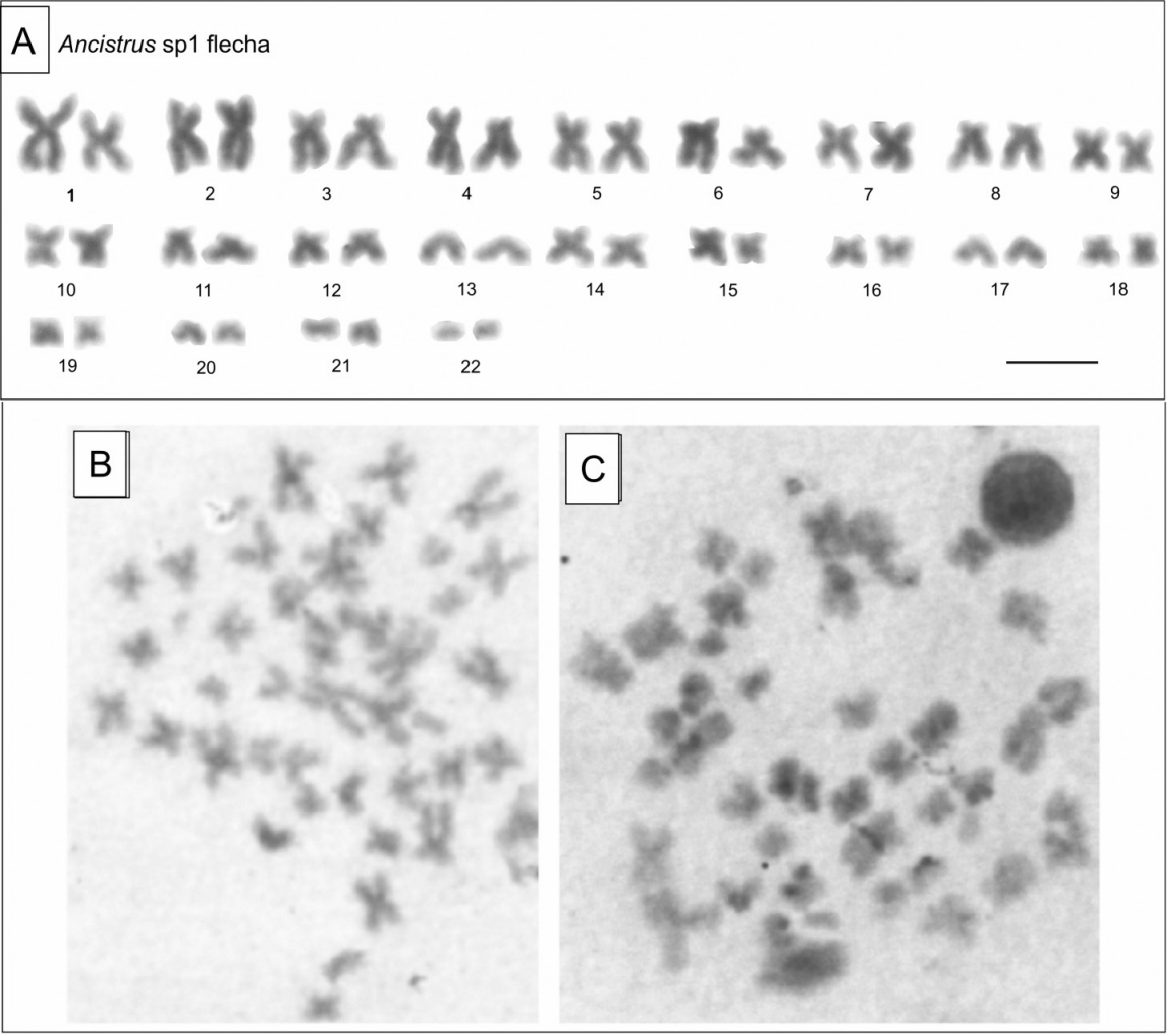
Karyotype of *Ancistrus* sp.1 “Flecha” using C-banding. **A** Conventional staining- showing the karyotype of *Ancistrus* sp.1 “Flecha” with 2n = 44 chromosomes, including a pair of microchromosomes **B, C** is C-band showing that the chromosomes of *Ancistrus* is not rich in heterochromatic regions in female and male. Bar = 10 µm.

**Figure 2.**
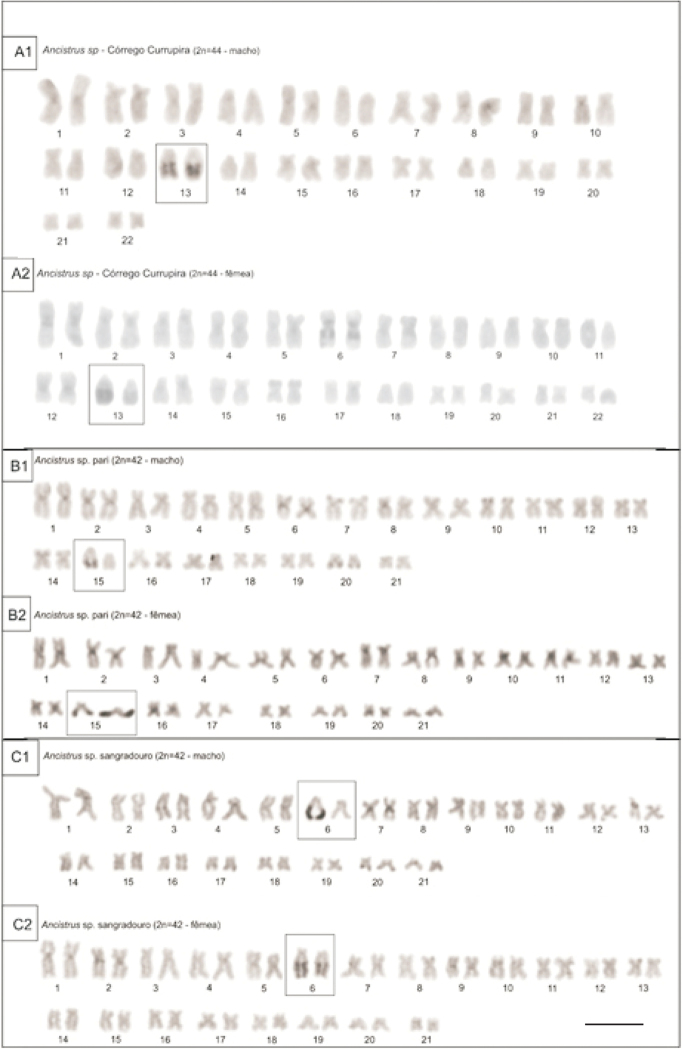
Karyotype after C band. *Ancistrus* sp. “Currupira” has 2n=44 chromosomes and showed heterochromatin blocks mainly at centromeric regions and pair 13 (**A**), *Ancistrus* sp. “Pari” has 2n=42 chromosomes with heterochromatin blocks along centromeric regions and a big block in pair 15 (**B**) and *Ancistrus* sp. “Sangradouro” has 2n=42 chromosomes, a karyotype similar to *Ancistrus* sp. “Pari” but the big heterochromatin block is in pair 6 (**C**). Bar = 10 µm.

The analysis of constitutive heterochromatin by C-banding was performed to characterize all species karyotypes covered in this study. *Ancistrus* sp.1 “Flecha” has 2n=44 chromosomes and no heterochromatin block or sex chromosome system was shown (Fig. 1). *Ancistrus* sp. “Currupira” has 2n=44 chromosomes and showed heterochromatin mainly at pericentromeric regions and a block in pair 13 (Fig. 2A), *Ancistrus* sp. “Pari” has 2n=42 chromosomes with heterochromatin along pericentromeric regions and a block in pair 15 (Fig. 2B) and *Ancistrus* sp.“Sangradouro” has 2n=42 chromosomes, a karyotype similar to *Ancistrus* sp. “Pari” but its heterochromatin block is in pair 6 (Fig. 2C).

### Analysis of AnDraI sequence

**Figure 3.**
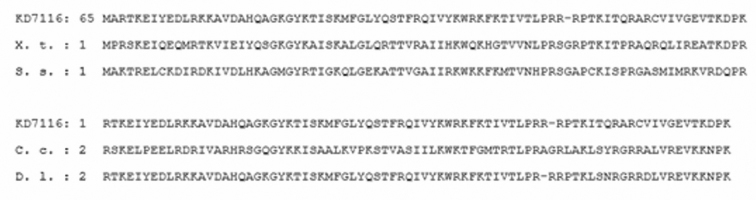
Comparison of the amino acid sequence of Transposase expected of AnDraI with Tc1-like transposase of other species. **X. t.** *Xenopus tropicalis* **S. s.** *Salmo salar* **C. c.** *Cyprinus carpio* **D. l.** *Dicentrarchus labrax*.

After isolation of repetitive sequences with restriction enzymes using *Ancistrus* sp.1 “Flecha” DNA, it was possible to observe the formation of a band of approximately 700 bp. The product from this band was then cloned, resulting in 34 recombinant clones, from which one, named AnDraI was used in this study. The sequence of this clone had 618 bp and 44.34% of GC base pair. According with the databases consulted: Blast 2.0 RepeatMasker and Censor, the sequence obtained showed greater than 86% identity with the type of transposon Mariner/Tc1 of *Xenopus tropicalis* (Gray, 1864). In the analysis performed for possible coding regions, an ORF region of frame 3+ 188 bp (87-275) was found. As conserved domain, a region of approximately 180 nucleotides which corresponds to HTH_Tnp_Tc3_2_Transposase was found. By submitting this sequence in the protein data bank (Blastx), similarities were found with transposases of several species, including, *Rana pipiens* (Schreber, 1782), *Xenopus tropicalis*, *Dicentrarchus labrax* (Linnaeus, 1758), *Salmo salar* (Linnaeus, 1758), and *Cyprinus carpio* (Linnaeus, 1758) (Fig. 3).

### PCR analysis of the sequence Tc1-like in specimens from other populations

It was realized an amplification of AnDraI element by polymerase chain reaction in the genome of other specimens of other populations: *Ancistrus* sp. “Currupira” from Currupira Creek, *Ancistrus* sp. “Pari” from Pari Creek and *Ancistrus* sp. “Sangradouro” from Sangradouro Creek. Both, males and females, of this species showed the same length of fragments (not shown).

### Chromosomal mapping of the transposon

**Figure 4.**
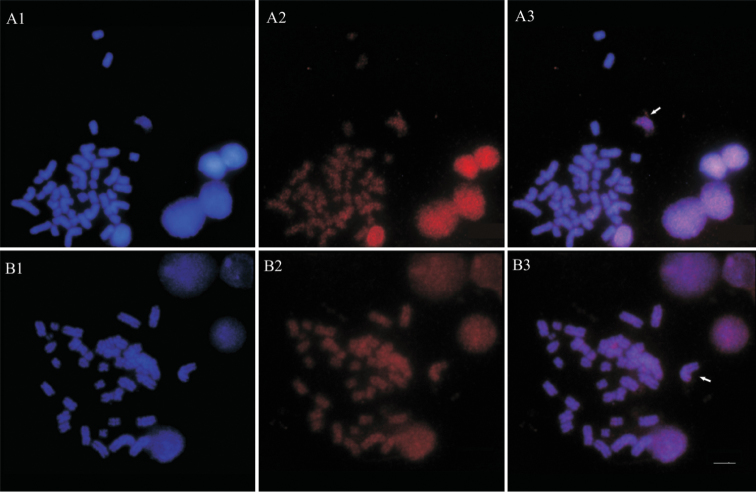
*In situ* fluorescence hybridization using AnDraI as probe in *Ancistrus* sp.1 “Flecha”: **A** female **B** male. The arrows show some examples of pericentromeric and spread signals. Bar = 10 µm.

Chromosomal *in situ* hybridization performed on *Ancistrus* sp.1 “Flecha” revealed signals throughout all the chromosomes preferentially located in pericentromeric regions, with no difference between males and females (Fig. 4).

**Figure 5.**
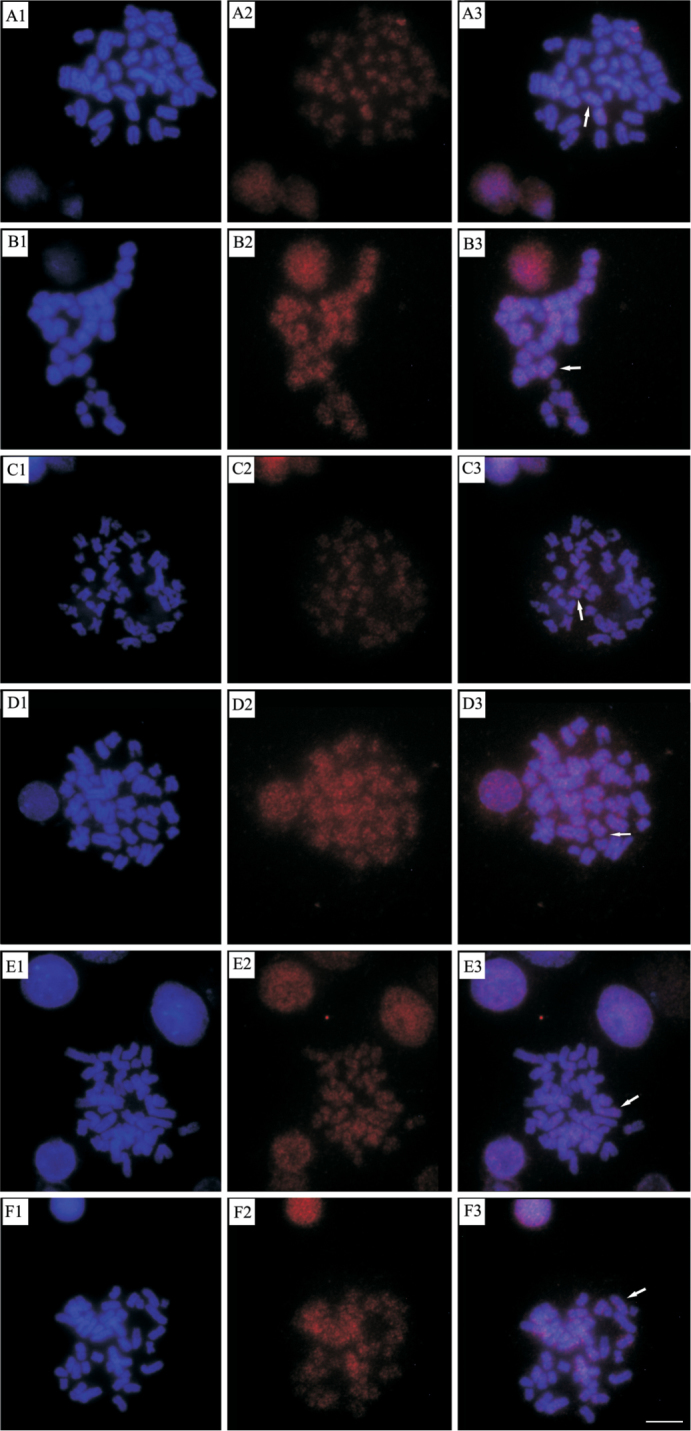
*In situ* fluorescence hybridization using AnDraI as probe in *Ancistrus* chromosomes: **A** Female of Currupira creek **B** Male of Currupira creek **C** Female of Pari creek **D** Male of Pari creek **E** Female of Sangradouro creek e **F** Male of Sangradouro creek. The arrows show some examples of pericentromeric and spread signals. Bar = 10 µm.

Cross-Fish conducted in individuals from other localities also showed results similar to that found in *Ancistrus* sp.1 “Flecha” (Fig. 5).

## Discussion

The transposable element Tc1, first identified in invertebrates as *Caenorhabditis elegans* (Maupas, 1900), have around 1600 bp and share similar structures, known as Terminal inversed regions (TIRs) that show a sequence of 5 to 6 bp identical in/or near the highly conserved ends (CAGTG/CAGTC) (Brezinsky et al. 1990, Avancini et al. 1996). However, there is a great variation between the number of copies, distribution and types of transposable elements, between different species arising from factors such as intrinsic characteristics of the transposable elements (TEs) and the different evolutionary forces that act on these (Capy et al. 1998).

The Tc1-like element isolated in this work from *Ancistrus* sp.1 “Flecha” appears to share a very similar structure to TcMar-Tc1 of *Xenopus* (Siluriana) *tropicalis*, which is 532 bp, equivalent to 86.1%. This element appears widely distributed throughout all the chromosomes of all specimens of *Ancistrus* covered in this study, with some specific concentrations in pericentromeric regions. These specimens belong to distinct localities with a high geographic distance and isolated among themselves, with different karyotypes which show number chromosomes ranging from 2n=42 to 44 and species with no sex chromosomes system and others with XX/XY or ZZ/ZW sex chromosomes. Despite of the known cytogenetic differences among the *Ancistrus* group it can be said that, at least among the specimens analyzed, the AnDraI element presents the same homogeneous pattern of distribution and no correlation can be made regarding the karyotypes differences neither the origin and differentiation of sex chromosomes of this group involving its isolated sequence. However, it can be inferred that, despite the low number of populations analyzed it is possible to consider that the AnDra I element is present in the genome of the *Ancistrus* genus. Although the Siluriformes group have a scientific and economic importance, their systematic and taxonomy are still highly problematic and, in this context, studies involving repetitive sequences, which are showed to be important cytogenetic markers, may help uncover the evolutionary history of the group.

Repetitive sequences may be present in centromeres and telomeres of eukaryotic chromosomes which are rich regions in heterochromatin, as well as regions over the interstitial chromosomal arms (Csink and Henikoff 1998). The *in situ* hybridization experiments in the chromosomes of *Ancistrus* sp. showed that the Tc1-like element is located throughout all the chromosomes with preferential markings in heterochromatic regions along the chromosomal arms, corroborating the results found in other studies carried out in other fish species, evidencing a pattern to disperse these elements (Capriglione et al. 2002, Ozouf-Costaz et al. 2004, Schemberger et al. 2014).

Results found in the literature about the genomic organization of transposons suggest that these elements are differently distributed in distinct groups of fish (Ferreira et al. 2011), as for example in the *Oreochromis niloticus* (Linnaeus, 1758) (Charlesworth et al. 1994, Martins 2007, Oliveira et al. 2013), Antartic Perciformes (Ozouf-Costaz et al. 2004) and in species of the subfamily Hypoptopomatinae (Dasilva et al. 2002), in which these elements can be found scattered throughout the genome. However, in species such as *Tetraodon nigroviridis* (Marion de Procé, 1822) (Dasilva et al. 2002, Fischer et al. 2004) and in most of the species of Cichlidae (Gross et al. 2009, Valente et al. 2011) they can be found accumulated in chromosomal regions rich in constitutive heterochromatin. Among the Siluriformes, the elements Rex1 and Rex3, in *Hisonotus leucofrenatu* (Ribeiro, 1908), *Pseudotocinclus tietensis* (Ihering, 1907) and *Parotocinclus maculicauda* (Steindachner, 1877), presented dispersed distributions patterns in the genome, similar to the pattern found for the transposable element AnDraI (Ferreira et al. 2011a), as well as a new dispersed element, BamHI, isolated by Ferreira et al. (2011b), in the genome of *Hisonotus leucofrenatus*. The genomic organization result of the Tc1-like element obtained in this work reinforces the hypothesis that the sequence AnDraI isolated is a dispersed element, and reinforces the hypothesis proposed by Ferreira et al. (2011) in which all transposable elements behave similarly inside of a family or subfamily. Also the study and characterization of these sequences can start to help to understand the evolutionary dynamics of *Ancistrus* genome, as well as the great karyotypic and chromosomal variability of this group, especially in the Paraguay river basin.
